# Trajectories of Healthcare Utilization Among Children and Adolescents With Autism Spectrum Disorder and/or Attention-Deficit/Hyperactivity Disorder in Japan

**DOI:** 10.3389/fpsyt.2021.812347

**Published:** 2022-01-20

**Authors:** Ai Aoki, Michi Niimura, Tsuguhiko Kato, Kenji Takehara, Junzo Iida, Takashi Okada, Tsunehiko Kurokami, Kengo Nishimaki, Kaeko Ogura, Masakage Okuno, Tatsuya Koeda, Takashi Igarashi, The Collaborative Working Group, Ayako Tamaoka

**Affiliations:** ^1^Department of Health Policy, National Center for Child Health and Development, Tokyo, Japan; ^2^Department of Social Medicine, National Center for Child Health and Development, Tokyo, Japan; ^3^Medical Faculty, Nara Medical University, Nara, Japan; ^4^Department of Developmental Disorders, National Center of Neurology and Psychiatry, Tokyo, Japan; ^5^Department of Psychosocial Medicine, National Center for Child Health and Development, Tokyo, Japan; ^6^National Rehabilitation Center for Persons With Disabilities, Saitama, Japan; ^7^Mikunihill Hospital, Osaka, Japan; ^8^National Center for Child Health and Development, Tokyo, Japan

**Keywords:** child and adolescent psychiatry, autism spectrum disorder (ASD), attention-deficit/hyperactivity disorder (ADHD), healthcare utilization, longitudinal analysis, early identification and intervention, Japan, neurodevelopmental disorders

## Abstract

**Background:**

Early intervention and prevention of psychiatric comorbidities of children with autism spectrum disorder (ASD) and attention-deficit/hyperactivity disorder (ADHD) are urgent issues. However, the differences in the diagnoses of ASD and ADHD and psychiatric comorbidities associated with age, long-term healthcare utilization trajectories, and its associated diagnostic features have not been fully elucidated in Japan.

**Method:**

We conducted a retrospective observational study using the medical records. Member hospitals of three major consortiums of hospitals providing child and adolescent psychiatric services in Japan were recruited for the study. Children who accessed the psychiatry services of the participating hospitals in April 2015 were followed up for 5 years, and data on their clinical diagnoses, consultation numbers, and hospitalizations were collected. Non-hierarchical clustering was performed using two 10-timepoint longitudinal variables: consultation numbers and hospitalization. Among the major clusters, the differences in the prevalence of ASD, ADHD, comorbid intellectual disability, neurotic disorders, and other psychiatric disorders were assessed.

**Results:**

A total of 44 facilities participated in the study (59.5%), and 1,003 participants were enrolled. Among them, 591 diagnosed with ASD and/or ADHD (58.9%) and 589 without missing data were assessed. The mean age was 10.1 years, and 363 (70.9%) were boys. Compared with the pre-schoolers, the school-aged children and adolescents had fewer ASD, more ADHD, and fewer comorbid intellectual disability diagnoses, as well as more diagnoses of other psychiatric disorders. A total of 309 participants (54.7%) continued consultation for 2 years, and 207 (35.1%) continued for 5 years. Clustering analysis identified three, two, and three major clusters among pre-schoolers, school-aged children, and adolescents, respectively. The largest cluster was characterized by early termination of the consultation and accounted for 55.4, 70.6, and 73.4% of pre-schoolers, school-aged children, and adolescents, respectively. Among the school-aged children, the diagnosis of ADHD was associated with a cluster that required longer periods of consultations. Among the adolescents, comorbid psychiatric disorders other than intellectual disability and neurotic disorders were associated with clusters that required hospitalization.

**Conclusion:**

Continuous healthcare needs were common and psychiatric comorbidities were associated with complex trajectory among adolescents. The promotion of early intervention and prevention of comorbidities are important.

## Introduction

Autism spectrum disorder (ASD) and attention-deficit/hyperactivity disorder (ADHD) are two common mental disorders in children and adolescents categorized as neurodevelopmental disorders (NDDs). ASD is characterized by social interaction and communication problems and restricted and repetitive patterns of behaviors, interests, or activities. ADHD is characterized by inattention, hyperactivity, and impulsivity (1). As high as 25.7–65.0% of people with ASD had comorbid ADHD (2). Based on the evidence that both are disorders with onset during the developmental phase and have considerable co-occurrence (3, 4), Diagnostic and Statistical Manual of Mental Disorders (DSM-5) allocates ASD and ADHD to the same category of NDDs (1). Previous studies have reported that the prevalence of ASD is ~1%, and the prevalence of ADHD is ~5% (5–7). As a reflection of the change in diagnostic criteria from the disease to the spectrum level and the increase in public awareness, in some countries, the reported prevalence of ASD and ADHD have been further increasing (8–12). In addition to their high prevalence, ASD and ADHD are important disorders, as ASD and ADHD often require continuous psychosocial and medical support.

The burden of ASD and ADHD on individuals, families, and society is huge (13–15). Children and adolescents with ASD or ADHD often experience psychiatric symptoms in addition to the core symptoms of ASD or ADHD (2, 16). Previous studies demonstrated that as high as 70% of adolescents with ASD and one-half of children and adolescents with ADHD have at least one psychiatric comorbidity (17, 18). To reduce the high burden of individuals with ASD and ADHD, early identification and support, including early developmental intervention and parental support, have been promoted in several high-income countries, such as the United States and the United Kingdom (19, 20). Early intervention is considered to reduce difficulties faced by individuals and help them improve their daily functioning and prevent the co-occurrence of psychiatric symptoms (13, 20, 21). However, some barriers against getting diagnosis of ASD and ADHD are known to exist such as lack of knowledge, stima, and poor service access (22, 23). In Japan, a large-scale school survey among primary and secondary school students demonstrated that 3.1% of students had inattention or hyperactivity problems and 1.1% had social interaction problems and restricted and repetitive patterns of behaviors, and they needed specialist assessment for suspicions of neurodevelopmental disorders in 2012. However, 40% of the participants did not receive any special support (24). There is an urgent need to promote early identification and intervention and prevent psychiatric comorbidities in children with ASD or ADHD in Japan (25).

Understanding the current healthcare utilization by children with ASD or ADHD is essential. However, the healthcare use trajectories have not been fully investigated in many countries including Japan. Instead, there had been some previous studies analyzing treatment dropout and hospitalization. Previous studies in the US identified that 30% of children with past ASD diagnosis and one fourth of children with past ADHD diagnosis did not receive current treatment (26, 27). Another study demonstrated that approximately 10% of children and young people with ASD require hospitalization during their treatment course and late diagnosis and comorbid psychiatric problems such as depression were risk factors for hospitalization (28). The evidence regarding the period of consultation, frequency of consultation over time and hospitalization and these patterns is still lacking.

In Japan, psychosocial development in children is assessed at health checkups at 1.5 and 3 years of age, as stipulated in the Maternal and Child Health Act (29). Children with developmental concerns were identified, followed up, and advised to get specialist assessment if necessary. Those who are overlooked in the screening process later visit specialists for assessment when they have greater concerns.

Therefore, the purpose of this study was to investigate age-related patterns of presentations, typical healthcare utilization trajectories, and associated diagnostic characteristics among children and adolescents with ASD and/or ADHD. It is essential to identify the clinical features of children with ASD and ADHD at these hospitals to improve their medical care and support in Japan.

## Methods

### Study Design

This was a retrospective medical record data analysis. The data were derived from the medical records of the patients. Children and adolescents who accessed the child and adolescent psychiatry outpatient services at participating hospitals for the first time in April 2015 were included in the survey. Healthcare utilization was followed up until March 2020; the 5 years were divided into 10 half-year periods. Participants with a primary or secondary diagnosis of ASD or ADHD were included in the analysis.

### Study Settings

The participating hospitals were members of the three major consortiums of hospitals providing child and adolescent psychiatric services in Japan. The three major consortiums are the child mental health network development program, the Japanese Association of Children's Hospitals and Related Institutions, and the Japanese Council of Child and Adolescent Mental Institution (30–32). The total number of hospital members was 74.

### Data Collection

The study data were collected and managed using the Research Electronic Data Capture hosted at National Center for Child Health and Development (33, 34). Research Electronic Data Capture is a secure web-based software platform designed to support data capture for research studies; it provides (1) an intuitive interface for validated data capture, (2) audit trails for tracking data manipulation and export procedures, (3) automated export procedures for seamless data downloads to common statistical packages, and (4) procedures for data integration and interoperability with external sources.

### Measures

Age, sex, and psychiatric diagnoses were collected. For each half-year, the number of outpatient consultations (continuous variable), the existence of hospitalization (binary variable), and the existence of multi-agency liaison (binary variable), such as educational or social welfare institutions, were collected. The duration of consultation was calculated using the first and last consultation dates during the study period. Psychiatric diagnoses were collected using the Tenth Revision of the International Classification of Diseases (ICD-10) (35). Regarding psychiatric disorders, pervasive developmental disorder (ICD-10: F84) was treated as an equivalent of ASD, and hyperkinetic disorder (ICD-10: F90) was treated as an equivalent of ADHD in the DSM-5 (36). Regarding psychiatric diagnoses other than ASD and ADHD, three categories were derived: (1) intellectual disabilities (ICD-10: F7), (2) neurotic, stress-related, and somatoform disorders (ICD-10: F4) as neurotic disorders, and (3) other psychiatric disorders other than intellectual disabilities and neurotic disorders as other psychiatric disorders. The diagnosis given prior to the visit to the participating hospitals and its age were not collected in this study.

### Statistical Analysis

The data were stratified by the three age groups according to the age of the first consultation: pre-school (<6 years old), school-aged (6 years old and more/ <10 years old), and adolescents [10 years old and more (37)]. A descriptive analysis was performed for each age group. The differences in the prevalence of ASD and ADHD and the three comorbidity categories for the three age groups were determined. Chi-squared test or Fisher's exact test was performed. Bonferroni adjustment for multiple comparisons was applied, and the statistical significance threshold was set at *p* = 0.016 (0.05/3).

### Cluster Analysis and Discriminant Analysis

Non-hierarchical clustering was performed on longitudinal data of two indicators: the number of consultations at the psychiatric outpatient service and the existence of hospitalization due to psychiatric problems. Although, psychiatric hospitalization rate was assumed low, children's psychiatric hospitalization implies high healthcare needs. Thus, hospitalization was also analyzed. The k-means method specifically designed for the longitudinal data was adopted as the clustering method (38). The number of clusters was set between 2 and 6, and the number of redraws was 50 for each cluster number. The clustering analysis is non-supervised learning and there is no gold standard method to determin the cluster number. In the present study, the number of clusters was determined using the cluster validity index (Calinski-Harabasz Score) and expert opinions. The stability of the model was assessed based on the consistency of the results of the complete dataset and the 80% subsample of the dataset. The subsample was randomly chosen 20 times. Analysis was conducted using R 3.6.2, klm3d package (39). Missing values were not imputed but the participants with missing values were excluded from the analysis. To examine the difference in diagnostic characteristics between the clusters, the difference between the major clusters that accounted for 5% or more of each age group was examined. Small clusters that accounted for <5% of each age group were removed from the statistical comparison, as the variability was not ignored. Differences in the prevalence of ASD, ADHD, comorbid intellectual disability, neurotic disorders, and other psychiatric disorders across the major clusters were examined using the chi-squared test or Fisher's exact test. Bonferroni adjustment was performed for multiple comparisons and set at *p* = 0.01 (0.05/5). Descriptive analysis was performed to demonstrate the clinical characteristics, such as 2-year consultation continuation, 5-year consultation continuation, consultation duration, hospitalization, and multi-agency liaison during the follow-up.

### Ethical Consideration

The opt-out procedure was performed before the data collection. This study was approved by the ethical committee of the National Center for Child Health and Development in Japan (2020-252).

## Results

### Participating Facilities and Participants

Forty-four facilities participated in this study (59.5%). A total of 1,003 participants visited the child and adolescent mental health services at the facilities for the first time in April 2015 and were eligible for the study. None of them refused to participate in the opt-out procedure, and all were included in the study. Among them, 591 were diagnosed with ASD and/or ADHD (58.9% of participants), and the data of 589 without missing values among the number of outpatient consultations and the existence of hospitalization were analyzed (99.7%). The mean age was 10.1 years (SD 4.2, pre-schoolers: 121; school-age, 160; adolescent, 308), and 363 (70.9%) were boys. A total of 470 (79.8%) and 207 (35.1%) participants were diagnosed with ASD and ADHD, respectively.

### Differences in Diagnosis Stratified by Age-Group

Of the pre-schoolers, 109 (90.1%) had ASD and 19 (15.7%) had ADHD. Of the school-aged children, 118 (73.8%) had ASD and 72 (45.0%) had ADHD. Of the adolescents, 243 (78.9%) had ASD and 116 (37.7%) had ADHD. There were statistically significant differences between the prevalence of ASD and ADHD among the pre-schoolers and older age groups (ASD pre-school vs. school-aged *P* = 0.001, pre-school vs. adolescent *P* = 0.01, ADHD pre-school vs. school-aged *P* < 0.001, pre-school vs. adolescent *P* < 0.001). The prevalence of intellectual disabilities was significantly lower among the school-aged children and adolescents than among the pre-schoolers (pre-school vs. school-aged *P* < 0.001, pre-school vs. adolescent *P* < 0.001), and that of other psychiatric disorders was significantly greater among school-aged children than among pre-schoolers (pre-school vs. school-aged *P* < 0.01, pre-school vs. adolescent *P* < 0.001); as high as 44.2, 59.1, and 59.3% of the pre-schoolers, school-aged children, and adolescents had at least one psychiatric comorbidity, respectively. Approximately 10% of patients had two or more psychiatric comorbidities (Table 1).

**Table 1:** Demographic and clinical background of participants in each age-group.

	**Pre-school** **(<6 years old)**	**School-aged (6 years old and more/ <10 years old)**	**Adolescent** **(10 years old and more)**	***P*-value**
*n*	121	160	308	
Age Mean (SD)	4.1 (1.1)	8.1 (1.1)	13.5 (2.3)	
Male *n* (%)	91 (75.2%)	122 (76.3%)	210 (68.2%)	Pre-school vs. school-aged: *P* = 0.95 School-aged vs. adolescent: *P* = 0.09 Pre-school vs. adolescent: *P* = 0.19
**Diagnosis**				
ASD *n* (%)	109 (90.1%)	118 (73.8%)	243 (78.9%)	Pre-school vs. school-aged: *P* = 0.001 School-aged vs. adolescent: *P* = 0.25 Pre-school vs. adolescent: *P* = 0.01
ADHD *n* (%)	19 (15.7%)	72 (45.0%)	116 (37.7%)	Pre-school vs. school-aged: *P* < 0.001 School-aged vs. adolescent: *P* = 0.15 Pre-school vs. adolescent: *P* < 0.001
**Psychiatric comorbidities**				
At least one comorbidity *n* (%)	53 (44.2%)	94 (59.1%)	182 (59.3%)	Pre-school vs. school-aged: *P* = 0.04 School-aged vs. adolescent: *P* = 1 Pre-school vs. adolescent: *P* = 0.02
2 and more comorbidities *n* (%)	14 (11.6%)	22 (13.8%)	35 (11.4%)	Pre-school vs. school-aged: *P* = 1 School-aged vs. adolescent: *P* = 1 Pre-school vs. adolescent: *P* = 1
Intellectual disabilities *n* (%)	38 (31.4%)	23 (14.4%)	34 (11.0%)	Pre-school vs. school-aged: *P* = 0.001 School-aged vs. adolescent: *P* = 0.37 Pre-school vs. adolescent: *P* < 0.001
Neurotic disorders *n* (%)	18 (14.9%)	28 (17.5%)	75 (24.4%)	Pre-school vs. school-aged: *P* = 0.67 School-aged vs. adolescent: *P* = 0.11 Pre-school vs. adolescent: *P* = 0.04
Other psychiatric disorders *n* (%)	2 (1.7%)	18 (11.3%)	43 (14.0%)	Pre-school vs. school-aged: *P* = 0.002 School-aged vs. adolescent: *P* = 0.50 Pre-school vs. adolescent: *P* < 0.001

*P-values are differences between age-groups were examined using the chi-squared test or Fisher's exact test*.

### Clinical Features of Each Age-Group

Consultations were continued for 319 participants (54.7%) for two or more years and 207 participants (35.1%) for 5 years. More than half of the participants continued consultations for two or more years. A total of 46 participants (7.8%) required hospitalization during the follow up period. Hospitalization rate was higher in older age groups.

Of these, 297 participants required any kind of multi-agency liaison at least once during the study period (50.4%). The most frequently collaborated agency was educational agency (*n* = 168, 28.5%), followed by social agencies (*n* = 127, 21.6%). The consultation duration, hospitalization and multi-agency liaison by age group were summarized in Table 2.

**Table 2:** Clinical features of participants in each age-group.

	**Pre-school (<6 years old)**	**School-Age 6 years old and more/ <10 years old)**	**Adolescent (10 years old and more)**
*n*	121	160	308
2-year consultation continuation *n* (%)	68 (57.1%)	96 (60.8%)	155 (50.7%)
5-year consultation continuation *n* (%)	43 (35.5%)	75 (46.9%)	89 (28.9%)
Hospitalization during the study period *n* (%)	2 (1.7%)	12 (7.5%)	32(10.4%)
**Multi-Agency liaison**			
Any agent *n* (%)	46 (38%)	89 (55.6%)	162 (52.6%)
Educational agent *n* (%)	21 (17.4%)	61 (38.1%)	86 (27.9%)
Social agent *n* (%)	28 (23.1%)	29 (18.1%)	70 (22.7%)
Abuse related *n* (%)	0 (0%)	5 (3.1%)	5 (1.6%)

### Results of Clustering

**Figure 1:**
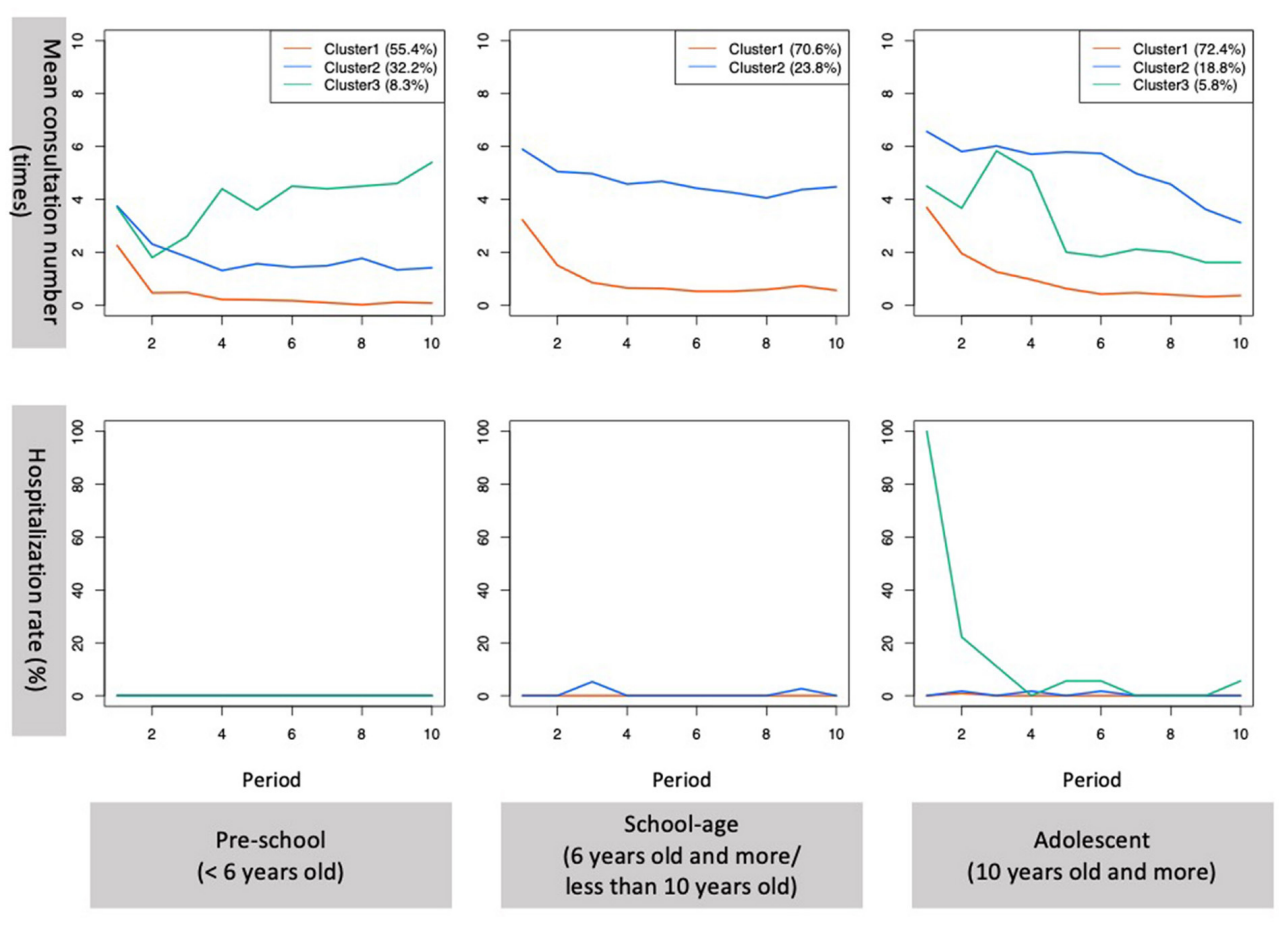
Mean consultation number and hospitalization rate per period for the major clusters. Period 1–10 indicate 10 consecutive half-year periods. Period 1 is April 2015 to September 2015 and period 10 is October 2019–March 2020. Only the trajectories of the major clusters in each age group are presented. Red, blue, and green lines indicate clusters 1, 2, and 3 for each group, respectively.

Clustering of healthcare utilization trajectories of pre-schoolers identified three major clusters and three small clusters. The first cluster (cluster 1) was characterized by early discontinuation of consultation after the first visit with an average length of consultation period of 1.2 years (SD 1.5) (*n* = 67, 55.4%). The second cluster (cluster 2) was characterized by stable continuous consultations at approximately once every 6 months (*n* = 39, 32.2%). The third cluster (cluster 3) was characterized by frequent consultations that increased over time (*n* = 10, 8.3%). During later periods, Cluster 2 required monthly consultations. No diagnosis was discriminative among the three clusters. Compared to Cluster 1, there was a trend that more participants in clusters 2 and 3 required multi-agency liaison. Small clusters were clusters that required intensive consultations at the beginning and clusters that required hospitalizations at some point during the follow-up [five participants (4.1%) in three small clusters, (Supplementary Tables 1, 2)] (Tables 3, 4, Figure 1).

**Table 3:** Clinical features of major clusters.

**Cluster**	**Participants**	**Duration of consultation (Years)**	**2-year continuation**	**5-year continuation**	**Hospitalization during study period**	**Outcome**		**Multi-Agency liaison**			
						**Agreed termination of the consultation**	**Referral to other health facilities**	**Any agent**	**Educational agent**	**Social agent**	**Abuse related**
	***n*** **(%)**	**Mean (SD)**	***n*** **(%)**	***n*** **(%)**	***n*** **(%)**	***n*** **(%)**	***n*** **(%)**	***n*** **(%)**	***n*** **(%)**	***n*** **(%)**	***n*** **(%)**
**Pre-school (<6 years old)**											
Cluster 1	67 (55.4%)	1.2 (1.5)	17 (25.8%)	6 (9.0%)	0 (0%)	25 (37.3%)	6 (9.0%)	20 (29.9%)	8 (11.9%)	13 (19.4%)	0 (0%)
Cluster 2	39 (32.2%)	4.2 (1.0)	36 (94.7%)	27 (69.2%)	0 (0%)	3 (7.7%)	4 (10.3%)	16 (41.0%)	8 (20.5%)	8 (20.5%)	0 (0%)
Cluster 3	10 (8.3%)	4.9 (0.0)	10 (100%)	9 (90.0%)	0 (0%)	0 (0%)	1 (10.0%)	6 (60.0%)	3 (30.0%)	5 (50.0%)	0 (0%)
**School-Aged (6 years old and more/ <10 years old)**											
Cluster 1	113 (70.6%)	2.4 (2.1)	55 (48.7%)	38 (33.6%)	0 (0%)	24 (21.2%)	13 (11.5%)	53 (46.9%)	34 (30.1%)	13 (11.5%)	0 (0%)
Cluster 2	38 (23.8%)	4.9 (0.1)	36 (100%)	33 (86.8%)	3 (7.9%)	0 (0%)	4 (10.5%)	27 (71.1%)	21 (55.3%)	10 (26.3%)	3 (7.9%)
**Adolescent (10 years old and more)**											
Cluster 1	223 (72.4%)	1.7 (1.9)	79 (35.6%)	37 (16.6%)	2 (0.9%)	62 (27.8%)	51 (22.9%)	99 (44.4%)	47 (21.1%)	37 (16.6%)	1 (0.4%)
Cluster 2	58 (18.8%)	4.5 (0.7)	57 (100%)	42 (72.4%)	3 (5.2%)	5 (8.6%)	10 (17.2%)	41 (70.7%)	25 (43.1%)	19 (32.8%)	2 (3.4%)
Cluster 3	18 (5.8%)	2.7 (2.0)	11 (61.1%)	5 (27.8%)	18 (100%)	2 (11.1%)	9 (50.0%)	16 (88.9%)	9 (50.0%)	9 (50.0%)	2 (11.1%)

**Table 4:** Diagnostic features of major clusters.

	**Demographics**				**Diagnosis**									
	**Age**		**Male**		**ASD**		**ADHD**		**Intellectual disabilities**		**Neurotic disorders**		**Other psychiatric disorders**	
**Cluster**	**Mean (SD)**	***P*-value**	***n* (%)**	***P*-value**	***n* (%)**	***P*-value**	***n* (%)**	***P*-value**	***n* (%)**	***P*-value**	***n* (%)**	***P*-value**	***n* (%)**	***P*-value**
**Pre-school (<6 years old)**														
Cluster 1	4.2 (1.1)	0.74	53 (79.1%)	0.37	60 (89.6%)	0.24	10 (14.9%)	0.39	19 (28.4%)	0.72	14 (20.9%)	0.18	0 (0%)	0.28
Cluster 2	4.0 (1.1)		27 (69.2%)		37 (94.9%)		5 (12.8%)		14 (35.9%)		3 (7.7%)		2 (5.1%)	
Cluster 3	4.3 (1.2)		9 (90.0%)		8 (80.0%)		3 (30.0%)		3 (30.0%)		1 (10.0%)		0 (0%)	
**School-Aged (6 years old and more/ <10 years old)**														
Cluster 1	8.1 (1.1)	0.98	82 (72.6%)	0.37	89 (78.8%)	0.04	42 (37.2%)	0.004	18 (15.9%)	0.60	20 (17.7%)	0.98	12 (10.6%)	0.77
Cluster 2	8.1 (1.1)		31 (81.6%)		23 (60.5%)		25 (65.8%)		4 (10.5%)		6 (15.8%)		5 (13.2%)	
**Adolescent (10 years old and more)**														
Cluster 1	13.5 (2.3)	0.71	156 (70.0%)	0.49	176 (78.9%)	0.64	84 (37.7%)	0.93	26 (11.7%)	0.80	51 (22.9%)	0.28	26 (11.7%)	0.005
Cluster 2	13.1 (2.3)		36 (62.1%)		45 (77.6%)		22 (37.9%)		5 (8.6%)		19 (32.8%)		7 (12.1%)	
Cluster 3	14.3 (2.5)		13 (72.2%)		16 (88.9%)		6 (33.3%)		2 (11.1%)		4 (22.2%)		7 (38.9%)	

*P-values are differences in the prevalence of ASD, ADHD, comorbid intellectual disability, neurotic disorders, and other psychiatric disorders across the major clusters were examined using the chi-squared test or Fisher's exact test*.

Among school-aged children, two major clusters and three small clusters were identified. The first cluster (cluster 1) was characterized by early discontinuation of consultation after the first visit with an average length of consultation period of 2.4 years (SD 2.1) (*n* = 113, 70.6%), and the second cluster (cluster 2) was characterized by stable continuous consultations at the monthly level (*n* = 38, 23.8%). Compared with Cluster 1, Cluster 2 had significantly more participants with ADHD (37.2 vs. 65.8%, *p* = 0.004). Compared with Cluster 1, more participants required multi-agency liaison, including abuse-related liaison, in Cluster 2 during the study period. Small clusters were clusters that required hospitalization at the beginning and during the follow-up and required frequent hospitalizations [nine subjects (5.6%) in three small clusters, (Supplementary Tables 1, 2)] (Tables 3, 4, Figure 1).

Among the adolescents, three major clusters and two small clusters were identified. The first cluster (cluster 1) was characterized by early discontinuation of consultation after the first visit with an average length of consultation of 1.7 years (SD 1.9) (*n* = 223, 72.4%). The second cluster (cluster 2) was characterized by continuous consultation (*n* = 58, 18.8%). The third cluster (cluster 3) was characterized by hospitalizations during the first 6 months after the first visit (*n* = 18, 5.8%). There was a significant difference in the prevalence of comorbid other psychiatric disorders among the major clusters (cluster 1 11.7%; cluster 2, 12.1%; cluster 3, 38.9%; *p* = 0.005). Compared to Cluster 1, more participants in Clusters 2 and 3 required multi-agency liaison. A higher proportion of participants in cluster 3 required multi-agency liaison because of child abuse. Small clusters were clusters that required hospitalization during the follow-up and frequent hospitalizations [nine participants (2.9%) in two small clusters, (Supplementary Tables 1, 2)] (Tables 3, 4, Figure 1).

### Stability of the Model

The stability of the clusters was assessed based on the stability of the results of 20 random sampling of 80% of the samples. The consistency was 97.4% for pre-schoolers, 98.4% for school-aged children, and 97.4% for adolescents.

## Discussion

### Summary of the Results

This study demonstrated that there are age-related diagnostic patterns among children with ASD or/and ADHD: ASD was dominant among pre-schoolers, while ADHD increased among school-aged children and adolescents. Regarding psychiatric comorbidities, intellectual disabilities decreased among children in older age groups, while neurotic disorders and other psychiatric disorders increased as they grew. A high prevalence of psychiatric comorbidities was observed. More than half of the study participants continued consultation for 2 years, and approximately one-third continued for 5 years. Approximately half of the participants required multi-agency liaison. For each age group, the largest cluster was a cluster that followed the simplest trajectories with features such as fewer consultations, shorter duration of consultation, and fewer to no hospitalizations. The largest cluster comprised approximately half of the pre-schoolers and 3/4 of the older age groups. Among the school-aged children, a more complex trajectory with long-lasting and frequent consultations was associated with the diagnosis of ADHD. Among adolescents, a more complex trajectory that required hospitalization was associated with comorbid psychiatric disorders other than intellectual disabilities and neurotic disorders. The proportion of participants who required multi-agency liaison during the treatment course was higher among children grouped into more complex healthcare use trajectory clusters.

Our results demonstrated that comorbid intellectual disability decreased in the older age groups. This is consistent with the evidence that neurodevelopmental problems in children without intellectual disabilities tend to be overlooked and diagnosed late (40). To support children with ASD and/or ADHD who manifest problems after entering primary school, strengthening school capacity and better collaboration between the health and education sectors is important. In Japan, this is promoted by allocating school nurses or other mental health specialists to counsel psychosocial and behavioral problems and collaborate with school doctors (41). To promote early identification, further screening for ASD and ADHD is important.

Regarding the features of clusters, our results demonstrated that the proportion of the largest cluster which was characterized by shorter duration of consultation and fewer to no hospitalization were larger among school-aged children and adolescents compared to pre-schoolers. This might reflect several points. First is that children with higher disability tend to visit hospital earlier so that pre-schoolers included more children with continuous medical needs. Second is that adolescents start to be referred to adult psychiatry services. In addition, there are other factors that associate with short duration of consultation such as parental parental compliance. However, our study did not investigate these points. Although 10–20% of children in the largest clusters were referred to other health facilities, majority of them terminated consultation. This result is consistent with the previous findings from the US that high proportion of children with past ASD or ADHD diagnosis did not receive current treatment (26, 27).

More participants in more complex healthcare use trajectories required multi-agency liaison. This implies that children with higher healthcare needs cannot be supported by medical services alone. Healthcare providers' recognition of the needs and health system's support for the promotion of multi-agency liaison is necessary. More complex healthcare use trajectories often involved hospitalization. The hospitalization rate among all participants (7.8%) was comparable to the previous findings about the hospitalization rate among children and young people with ASD from the US (10.8%) (28).

In our study, there was an age-associated increasing trend in psychiatric comorbidities other than intellectual disabilities. This is consistent with the results of a systematic review that demonstrated that the prevalence of various comorbid disorders increased with older age compared with the prevalence of ASD among adolescents and a review that demonstrated that the prevalence of comorbid depression increased in older children with ASD (2, 16, 42). The clustering analysis demonstrated that psychiatric comorbidities were associated with a more complex trajectory that required hospitalization among adolescents. This result implies that adolescents with ASD and/or ADHD with psychiatric comorbidities other than intellectual disability and neurotic disorder tend to have more healthcare needs, and there is a need to promote early identification and ensure service access in order to prevent children with ASD and ADHD from developing psychiatric comorbidities.

### Limitations

First, this was a retrospective observational study that used medical records. Thus, our dataset does not include any clinical severity variables or socioeconomic variables associated with healthcare utilization. Nor, our study did not collect data on the changes in diagnosis during the treatment course. The results serve as a foundation for future research to investigate the factors that identify the target of early intervention or intensive intervention, and prospective longitudinal research is needed in this field in the future.

Second, the sample size was not sufficient to capture less frequent trajectories or differences in diagnostic patterns between the age groups. From our results, the less frequent trajectories involved frequent consultations and hospitalizations and were considered more complex. With a larger sample size, the association between less frequent trajectories and diagnosis may be significant. The difference in the proportion of comorbidities between school-aged children and adolescents may be considered significant with a larger sample size.

Third, this study could not follow healthcare utilization in health facilities other than the enrolled hospitals. However, child and adolescent psychiatry service is relatively centralized in Japan. The hospitals enrolled in the current study play a central role in child and adolescent psychiatry within their catchment areas. It could be assumed that participants with high healthcare needs had continued consultations at the same hospital.

Finally, as our study enrolled hospitals belonging to three major consortiums, the participation rate was ~60%, and population representativeness was not high. However, as this was a retrospective medical record survey, there was no drop-out or refusal to participate in the opt-out procedure, which contributed to better data quality.

## Conclusion

This study demonstrated age-related changes in diagnostic patterns among children with ASD or ADHD and their typical healthcare use trajectories. Future research investigating the factors associated with complex trajectories is necessary. The results imply that early identification, intervention, and prevention of psychiatric comorbidities and support involving multi-agency liaison are crucial, and they need to be promoted.

## Data Availability Statement

The raw data supporting the conclusions of this article will be made available by the authors, without undue reservation.

## Ethics Statement

The studies involving human participants were reviewed and approved by the Ethical Committee of the National Center for Child Health and Development in Japan. Written informed consent from the participants' legal guardian/next of kin was not required to participate in this study in accordance with the national legislation and the institutional requirements.

## Author Contributions

AA, TKa, KT, JI, TO, TKu, KN, KO, MO, TKo, and TI developed the concept of the study. JI, TO, TKu, KN, KO, MO, TKo, and TI coordinated the logistics, including the recruitment of the participating hospitals. MN created a data collection platform and supervised the data collection. The collaborative group members coordinated the logistics at each hospital and conducted data collection. AA, MN, TKa, and KT analyzed the data. AA drafted the manuscript. All authors critically reviewed the manuscript and approved the final version of the manuscript.

## The Collaborative Working Group Members

Ayako Tamaoka, Department of Psychiatry, Hyogo Prefectural Kobe Children's Hospital, Kobe, Japan; Fumio Matsuda, Matsuda Hospital, Kobe, Japan; Hideo Honda, Mental Health Clinic for Children, Shinshu University Hospital, Matsumoto, Japan; Hideto Kanda, Yamagata Prefectural Mental Care Center, Yamagata, Japan; Hidetoshi Takahashi, Kochi Medical School Hospital, Nankoku, Japan; Hiroshi Yamashita, Department of Child Psychiatry, Kyushu University Hospital, Fukuoka, Japan; Jun-ichi Yamamura, Department of Child and Adolescent Psychiatry, National Hospital Organization Tenryu Hospital, Hamamatsu, Japan; Junko Tomita, Tohoku Fukushi University Sendan Hospital, Sendai, Japan; Kakurou Aoyagi, Yamanashi Prefectural Akebono Medical Welfare Center, Yamagata, Japan; Kanami Maegawa, Osaka Women's and Children's Hospital, Izumi, Japan; Kazuhiro Muramatsu, Department of Pediatrics, Jichi Medical University, Shimotsuke, Japan; Kazunori Makino, Saitama Prefectural Psychiatric Hospital, Saitama, Japan; Kei Akamatsu, Miyazakihigashi National Hospital, Miyazaki, Japan; Keiko Deguchi, Yamanashi Prefectural Center For Psychological Development, Kofu, Japan; Kiwamu Tanaka, Hyogo Mental Health Center, Kobe, Japan; Koichi Maruyama, Department of Pediatric Neurology, Aichi Developmental, Disability Center Central Hospital, Kasugai, Japan; Kozo Ohcho, Okayama Psychiatric Medical Center, Okayama, Japan; Kumi Inazaki, Kohnodai Hospital, National Center for Global Health and Medicine, Tokyo, Japan; Maho Hasebe, Yamanashi Prefectural KITA Hospital, Nirasaki, Japan; Mari Kasahara, Komagino Hospital, Hachioji, Japan; Masami Hanafusa, Osaka Psychiatric Medical Center, Hirakata, Japan; Miyuki Ushida, Department of Psychosomatic Medicine and Psychiatry for Children and Adolescents, National Hospital Organization Shikoku Medical Center for Children and Adults, Zentsuji, Japan; Ryo Sumazaki, Ibaraki Children's Hospital, Mito, Japan; Sakiho Ando, Chiba Children's Hospital, Chiba, Japan; Satoshi Harada, National Hospital Organization Ryukyu Hospital, Kin, Japan; Shin-ya Iida, Child and Adolescent Psychiatry, Osaka City General Hospital, Osaka, Japan; Takaaki Abe, Jichi Children's Medical Center Tochigi, Shimotsuke, Japan; Takafumi Kobayashi, Shimane Prefectural Psychiatric Medical Center, Izumo Japan; Takashi Arai, Department of child and adolescent psychiatry, Kanagawa Children's Medical Center, Yokohama, Japan; Takuya Saito, Department of Child and Adolescent Psychiatry, Hokkaido University Hospital, Sapporo, Japan; Tatsuru Kitamura, Ishikawa Prefectural Takamatsu Hospital, Kahoku, Japan; Tomoatsu Isono, Department of Psychiatry and Child Psychiatry, Asahi General Hospital, Asahi, Japan;Toru Yoshikawa, Department of Child Psychiatry, Aichi Developmental Disability Center Central Hospital, Kasugai, Japan; Tsuyoshi Matsuoka, Division of Child Neurology and Child Psychiatry, Okinawa Prefectural Nanbu Medical Center and Children 's Medical Center, Haebaru, Japan; Tsuyoshi Sasaki, Department of Child Psychiatry, Chiba-University Hospital, Chiba, Japan; Yasuhisa Seguchi, Hizen Psychiatric Medical Center, Yoshinogari, Japan; Yokota Shingo, Hannan Hospital, Sakai, Japan; Yoshihiro Maegaki, Division of Child Neurology, Institute of Neurological Sciences, Faculty of Medicine, Tottori University, Tottori, Japan; Yoshihiro Nakadoi, Department of Child Psychiatry, Shikoku Medical Center for Children and Adults, Zentsuji, Japan; Yugo Miyata, Medical Corporation Camellia Oomurakyoritsu Hospital, Nagasaki, Japan; Yukiko Kano, Department of Child Psychiatry, The University of Tokyo Hospital, Tokyo, Japan; Yurika Numata-Uematsu, Department of Pediatrics, Tohoku University School of Medicine, Sendai, Japan; Yuzuru Harada, Nagano Prefectual Mental Wellness Center-Komagane, Komagane, Japan.

## Funding

This study was supported by the Japanese Ministry of Health, Labor, and Welfare (Grant No. 20GC1019). The funding body did not have any roles in the design of this study, data collection, data analysis, interpretation of results, or writing of the manuscript.

## Conflict of Interest

The authors declare that the research was conducted in the absence of any commercial or financial relationships that could be construed as a potential conflict of interest.

## Publisher's Note

All claims expressed in this article are solely those of the authors and do not necessarily represent those of their affiliated organizations, or those of the publisher, the editors and the reviewers. Any product that may be evaluated in this article, or claim that may be made by its manufacturer, is not guaranteed or endorsed by the publisher.

## Abbreviations

ADHD: Attention-deficit/hyperactivity disorder
ASD: Autism spectrum disorder
ICD: International Classification of Diseases
NDD: Neurodevelopmental disorder.
