# Supplementary material for: Trajectories of Healthcare Utilization Among Children and Adolescents With Autism Spectrum Disorder and/or Attention-Deficit/Hyperactivity Disorder in Japan
Source: Front Psychiatry. 2022 Jan 20;12:812347. doi: 10.3389/fpsyt.2021.812347 (PMC8811216; doi:10.3389/fpsyt.2021.812347)
Supplement: Supplementary file 1 [file Table_1.DOCX]

**Supplementary table 1. Clinical features of all clusters**

|  | Participants | Duration of consultation (Years) | 2-year continuation | 5-year continuation | Hospitalization during study period | Outcome | | Multi-agency liaison | | | |
| --- | --- | --- | --- | --- | --- | --- | --- | --- | --- | --- | --- |
|  |  |  |  |  |  | Agreed termination of the consultation | Referral to other health facilities | Any agent | Educational agent | Social agent | Abuse related |
| Cluster | n (%) | Mean (SD) | n (%) | n (%) | n (%) | n (%) | n (%) | n (%) | n (%) | n (%) | n (%) |
| Preschool (<6 years old) | | | | | | | | | | | |
| All | 121 (100%) | 2.6 (2.0) | 68 (57.1%) | 43 (35.5%) | 2 (1.7%) | 29 (24%) | 13 (10.7%) | 46 (38%) | 21 (17.4%) | 28 (23.1%) | 0 (0%) |
| Major clusters | | | | | | | | | | | |
| Cluster 1 | 67 (55.4%) | 1.2 (1.5) | 17 (25.8%) | 6 (9.0%) | 0 (0%) | 25 (37.3%) | 6 (9.0%) | 20 (29.9%) | 8 (11.9%) | 13 (19.4%) | 0 (0%) |
| Cluster 2 | 39 (32.2%) | 4.2 (1.0) | 36 (94.7%) | 27 (69.2%) | 0 (0%) | 3 (7.7%) | 4 (10.3%) | 16 (41.0%) | 8 (20.5%) | 8 (20.5%) | 0 (0%) |
| Cluster 3 | 10 (8.3%) | 4.9 (0.0) | 10 (100%) | 9 (90.0%) | 0 (0%) | 0 (0%) | 1 (10.0%) | 6 (60.0%) | 3 (30.0%) | 5 (50.0%) | 0 (0%) |
| Small clusters | | | | | | | | | | | |
| Cluster 4 | 3 (2.5%) | 3.4 (0.4) | 3 (100%) | 0 (0%) | 0 (0%) | 1 (33.3%) | 1 (33.3%) | 2 (66.7%) | 2 (66.7%) | 0 (0%) | 0 (0%) |
| Cluster 5 | 1 (0.8%) | 4.9 (NA) | 1 (100%) | 0 (0%) | 1 (100%) | 0 (0%) | 1 (100%) | 1 (100%) | 0 (0%) | 1 (100%) | 0 (0%) |
| Cluster 6 | 1 (0.8%) | 4.9 (NA) | 1 (100%) | 1 (100%) | 1 (100%) | 0 (0%) | 0 (0%) | 1 (100%) | 0 (0%) | 1 (100%) | 0 (0%) |
| School-aged (6 years old and more/ less than 10 years old) | | | | | | | | | | | |
| All | 160 (100%) | 3 (2.1) | 96 (60.8%) | 75 (46.9%) | 12 (7.5%) | 24 (15%) | 19 (11.9%) | 89 (55.6%) | 61 (38.1%) | 29 (18.1%) | 5 (3.1%) |
| Major clusters | | | | | | | | | | | |
| Cluster 1 | 113 (70.6%) | 2.4 (2.1) | 55 (48.7%) | 38 (33.6%) | 0 (0%) | 24 (21.2%) | 13 (11.5%) | 53 (46.9%) | 34 (30.1%) | 13 (11.5%) | 0 (0%) |
| Cluster 2 | 38 (23.8%) | 4.9 (0.1) | 36 (100%) | 33 (86.8%) | 3 (7.9%) | 0 (0%) | 4 (10.5%) | 27 (71.1%) | 21 (55.3%) | 10 (26.3%) | 3 (7.9%) |
| Small clusters | | | | | | | | | | | |
| Cluster 3 | 5 (3.1%) | 1.3 (1.1) | 1 (20.0%) | 0 (0%) | 5 (100%) | 0 (0%) | 2 (40.0%) | 5 (100%) | 3 (60.0%) | 3 (60.0%) | 0 (0%) |
| Cluster 4 | 3 (1.9%) | 4.9 (0.1) | 3 (100%) | 3 (100%) | 3 (100%) | 0 (0%) | 0 (0%) | 3 (100%) | 2 (66.7%) | 2 (66.7%) | 1 (33.3%) |
| Cluster 5 | 1 (0.6%) | 4.9 (NA) | 1 (100%) | 1 (100%) | 1 (100%) | 0 (0%) | 0 (0%) | 1 (100%) | 1 (100%) | 1 (100%) | 1 (100%) |
| Adolescent (10 years old and more) | | | | | | | | | | | |
| All | 308 (100%) | 2.4 (2.1) | 155 (50.7%) | 89 (28.9%) | 32 (10.4%) | 70 (22.7%) | 73 (23.7%) | 162 (52.6%) | 86 (27.9%) | 70 (22.7%) | 5 (1.6%) |
| Major clusters | | | | | | | | | | | |
| Cluster 1 | 223 (72.4%) | 1.7 (1.9) | 79 (35.6%) | 37 (16.6%) | 2 (0.9%) | 62 (27.8%) | 51 (22.9%) | 99 (44.4%) | 47 (21.1%) | 37 (16.6%) | 1 (0.4%) |
| Cluster 2 | 58 (18.8%) | 4.5 (0.7) | 57 (100%) | 42 (72.4%) | 3 (5.2%) | 5 (8.6%) | 10 (17.2%) | 41 (70.7%) | 25 (43.1%) | 19 (32.8%) | 2 (3.4%) |
| Cluster 3 | 18 (5.8%) | 2.7 (2.0) | 11 (61.1%) | 5 (27.8%) | 18 (100%) | 2 (11.1%) | 9 (50.0%) | 16 (88.9%) | 9 (50.0%) | 9 (50.0%) | 2 (11.1%) |
| Small clusters | | | | | | | | | | | |
| Cluster 4 | 8 (2.6%) | 4 (1.3) | 7 (87.5%) | 4 (50.0%) | 8 (100%) | 1 (12.5%) | 3 (37.5%) | 5 (62.5%) | 5 (62.5%) | 4 (50.0%) | 0 (0%) |
| Cluster 5 | 1 (0.3%) | 4.9 (NA) | 1 (100%) | 1 (100%) | 1 (100%) | 0 (0%) | 0 (0%) | 1 (100%) | 0 (0%) | 1 (100%) | 0 (0%) |
