# Supplementary material for: Trajectories of Healthcare Utilization Among Children and Adolescents With Autism Spectrum Disorder and/or Attention-Deficit/Hyperactivity Disorder in Japan
Source: Front Psychiatry. 2022 Jan 20;12:812347. doi: 10.3389/fpsyt.2021.812347 (PMC8811216; doi:10.3389/fpsyt.2021.812347)
Supplement: Supplementary file 2 [file Table_2.docx]

**Supplementary table 2. Diagnostic features of all clusters**

|  | Demographics | | Diagnosis | | | | |
| --- | --- | --- | --- | --- | --- | --- | --- |
|  | Age | Male | ASD | ADHD | Intellectual disabilities | Neurotic disorders | Other psychiatric disorder |
| Cluster | Mean (SD) | n (%) | n (%) | n (%) | n (%) | n (%) | n (%) |
| Preschool (<6 years old) | | | | | | | |
| All | 4.1 (1.1) | 91 (75.2%) | 109 (90.1%) | 19 (15.7%) | 38 (31.4%) | 18 (14.9%) | 2 (1.7%) |
| Major clusters | | | | | | | |
| Cluster 1 | 4.2 (1.1) | 53 (79.1%) | 60 (89.6%) | 10 (14.9%) | 19 (28.4%) | 14 (20.9%) | 0 (0%) |
| Cluster 2 | 4 (1.1) | 27 (69.2%) | 37 (94.9%) | 5 (12.8%) | 14 (35.9%) | 3 (7.7%) | 2 (5.1%) |
| Cluster 3 | 4.3 (1.2) | 9 (90.0%) | 8 (80.0%) | 3 (30.0%) | 3 (30.0%) | 1 (10.0%) | 0 (0%) |
| Small clusters | | | | | | | |
| Cluster 4 | 3.4 (0.7) | 2 (66.7%) | 3 (100%) | 0 (0%) | 2 (66.7%) | 0 (0%) | 0 (0%) |
| Cluster 5 | 4 (NA) | 0 (0%) | 0 (0%) | 1 (100%) | 0 (0%) | 0 (0%) | 0 (0%) |
| Cluster 6 | 3 (NA) | 0 (0%) | 1 (100%) | 0 (0%) | 0 (0%) | 0 (0%) | 0 (0%) |
| School-aged (6 years old and more/ less than 10 years old) | | | | | | | |
| All | 8.1 (1.1) | 122 (76.3%) | 118 (73.8%) | 72 (45.0%) | 23 (14.4%) | 28 (17.5%) | 18 (11.3%) |
| Major clusters | | | | | | | |
| Cluster 1 | 8.1 (1.1) | 82 (72.6%) | 89 (78.8%) | 42 (37.2%) | 18 (15.9%) | 20 (17.7%) | 12 (10.6%) |
| Cluster 2 | 8.1 (1.1) | 31 (81.6%) | 23 (60.5%) | 25 (65.8%) | 4 (10.5%) | 6 (15.8%) | 5 (13.2%) |
| Small clusters | | | | | | | |
| Cluster 3 | 8.8 (1.1) | 5 (100%) | 4 (80.0%) | 3 (60.0%) | 0 (0%) | 1 (20.0%) | 1 (20.0%) |
| Cluster 4 | 8.7 (1.1) | 3 (100%) | 1 (33.3%) | 2 (66.7%) | 1 (33.3%) | 1 (33.3%) | 0 (0%) |
| Cluster 5 | 7.9 (NA) | 1 (100%) | 1 (100%) | 0 (0%) | 0 (0%) | 0 (0%) | 0 (0%) |
| Adolescent (10 years old and more) | | | | | | | |
| All | 13.5 (2.3) | 210 (68.2%) | 243 (78.9%) | 116 (37.7%) | 34 (11.0%) | 75 (24.4%) | 43 (14.0%) |
| Major clusters | | | | | | | |
| Cluster 1 | 13.5 (2.3) | 156 (70.0%) | 176 (78.9%) | 84 (37.7%) | 26 (11.7%) | 51 (22.9%) | 26 (11.7%) |
| Cluster 2 | 13.1 (2.3) | 36 (62.1%) | 45 (77.6%) | 22 (37.9%) | 5 (8.6%) | 19 (32.8%) | 7 (12.1%) |
| Cluster 3 | 14.3 (2.5) | 13 (72.2%) | 16 (88.9%) | 6 (33.3%) | 2 (11.1%) | 4 (22.2%) | 7 (38.9%) |
| Small clusters | | | | | | | |
| Cluster 4 | 14.2 (2.5) | 5 (62.5%) | 5 (62.5%) | 4 (50.0%) | 1 (12.5%) | 0 (0%) | 3 (37.5%) |
| Cluster 5 | 18.6 (NA) | 0 (0%) | 1 (100%) | 0 (0%) | 0 (0%) | 1 (100%) | 0 (0%) |
